# Effect of a Mentor Mother Programme on retention of mother-baby pairs in HIV care: A secondary analysis of programme data in Uganda

**DOI:** 10.1371/journal.pone.0223332

**Published:** 2019-10-14

**Authors:** Jude Ofuzinim Igumbor, Joseph Ouma, Kennedy Otwombe, Eustasius Musenge, Felix Chima Anyanwu, Tariro Basera, Marjorie Mbule, Esca Scheepers, Kathrin Schmitz

**Affiliations:** 1 School of Public Health, Faculty of Health Sciences, University of the Witwatersrand, Johannesburg, South Africa; 2 Perinatal HIV Research Unit, Faculty of Health Sciences, University of the Witwatersrand, Johannesburg, South Africa; 3 mothers2mothers, Cape Town, South Africa; University of Ghana College of Health Sciences, GHANA

## Abstract

**Background:**

Community healthcare workers (CHWs) play an important role in promoting HIV-care retention. Notwithstanding inconsistencies in the outcomes of CHW programmes, these programmes are known to have a positive effect on retention of mother-baby pairs in HIV-care in sub-Saharan Africa.

**Aim:**

The aim of this analysis was to assess the effect of mothers2mothers (m2m) Ugandan Mentor Mother (MM) programme on the retention of mother-baby pairs in HIV-care.

**Methods:**

We conducted a secondary analysis of data obtained from the m2m Uganda MM programme in nine East Central districts. The primary data was generated through a quasi-experimental study of women attending prevention of mother to child transmission of HIV (PMTCT) clinics in Uganda between January 2011 and March 2014; where those who were enrolled at PMTCT sites with the MM intervention (n = 1161) were compared with those who received standard PMCTCT services without the MM intervention (n = 1143). Frequencies and descriptive statistics were calculated for categorical and continuous measures respectively. Risk factors for retention in care were determined by clustered generalised estimating equations and reported as adjusted odds ratios (AOR) with 95% confidence intervals (95% CI).

**Results:**

Retention in the PMTCT cascade was significantly higher for mother-baby pairs in the intervention arm compared to those in the control arm across all measured time points (96.7% vs 65.8% at 6 weeks after birth, p<0.001; 81.5% vs 42% at 6 weeks after cessation of breastfeeding, p<0.001; and 71.2% vs 20.6% at 18 months after birth, p<0.001). Relative to the control group, women in the intervention group were less likely to be lost to follow up following treatment initiation (AOR 0.05, 95% CI: 0.02, 0.15). There was no difference in the proportion of the retained mother-baby pairs who received prescribed PMTCT interventions at different time points but a significantly higher number of mother-baby pairs in the intervention arm were retained at different time points.

**Conclusion:**

HIV positive mothers and their HIV exposed children in the mothers2mothers Ugandan Mentor Mother programme had higher retention in HIV care at every step along the PMTCT cascade. We therefore recommend adoption of this peer-to-peer model in sub-Saharan Africa to complement retention in care strategies and health system interventions especially among priority and key populations.

## Introduction

In 2013, Uganda introduced Option B+, wherein all HIV-positive pregnant women and breastfeeding mothers were started on lifelong antiretroviral therapy (ART) regardless of their CD4 count [1]. Four years after implementing this strategy, 3 637 health facilities were strengthened to provide ART to pregnant and breastfeeding women living with HIV [2]. As a result more than 97% of HIV-positive pregnant women received ART in 2016 alone [3]. However, successful engagement along the HIV treatment cascade remains a major concern among mother-baby pairs, this is particularly the case in low-income countries (LICs) where studies have reported instances of, about 81% of mother-baby pairs who are not retained in care six months after delivery [4,5]. To verify these findings, studies in Uganda have reported attrition rates in mother-baby pairs on Option B+, ranging from 34.7% [6] to 70% [7]. As suggested by the Uganda AIDS Commission, only 38% of HIV exposed infants are available for final HIV testing at 18 months and this has been attributed to low retention of mother-baby pairs in HIV care [8]. Non-retention of HIV exposed infants increases their risk of infection resulting from infants not having access to uninterrupted prophylactic treatment and monitoring, secondary prevention messaging and support, and opportunities for prompt diagnosis and treatment as their HIV status will remain unknown [1,6,9].

Lifelong retention in HIV-care is vital to the successful prevention and elimination of mother to child transmission of HIV (PMTCT/eMTCT). For this reason, improving and sustaining survival of mothers and their infants will require identification and implementation of effective, feasible, and scalable strategies to engage and retain HIV-positive pregnant women, and postnatal mothers and their infants in HIV-care and treatment programs across the PMTCT/ HIV care cascade [10]. However, there are known barriers that impede the retention of mother-child pair in HIV care; which include overcrowded clinics, long waiting times, lack of privacy during counselling sessions, transportation costs, drug side effects, stigma, depression and inadequate social support [6,11,12]. Furthermore, studies have associated poor retention with patients in rural settings, women under 25 years of age and those attending care at lower level health facilities [1,13]. In addition, long term discontinuation of care is mostly attributed to psychological and social barriers, whereas structural barriers contribute to more silent or undocumented transfers of patients [12].

Conversely, improved retention has been associated with interventions such as peer mentoring and counselling, psychosocial support, mobile phone calls and text messages, home follow-up visits, and the integration of PMTCT into routine health care [14–16]. Yet, providing such level of care and support is fraught with a number of challenges ranging from shortage of healthcare workers to overburdened health systems [17]. Strikingly, empirical analysis of relevant literature favours arguments that shortage of healthcare workers can be addressed by task shifting of health services from highly qualified and trained health professionals to health workers with fewer qualifications and less training. This has created a new era of ancillary health workers support system [10]. Further evidence suggests that this could be achieved through community-based interventions such as engaging community health workers (CHWs) or peer educators, who are known to promote ongoing adherence support and monitoring of patients at the community. Such strategies would ensure that HIV infected mother-baby pairs remain engaged in care through home visits, peer mentoring, counselling and psychosocial support [14–16].

Providing this kind of support is the cardinal goal of the mothers2mothers (m2m) initiative, a Non-Governmental Organization (NGO) that is based in Africa[18]. This organisation has been in operation since 2001, originally working closely with different stakeholders to eliminate paediatric HIV and AIDS. Although m2m’s initial focus was to eliminate mother-to-child transmission of HIV, it has expanded since 2014 to combat the causes and effects of HIV/AIDS in a more comprehensive manner. For instance, while eliminating paediatric AIDS has remained at the heart of organisational efforts, the organisational approach encompasses the full cycle of life from pregnancy, through infancy, to childhood and adolescence. In addition, the m2m model is to employ HIV-positive women as paid, professionalised community health workers and peer mentors. Peer mentors or “Mentor Mothers” are integrated into the formal healthcare system and work in both health facilities and in local communities. They ensure that women, their families, and their communities are effectively engaged in care and assessed, referred and linked to appropriate clinical services; and supported on their treatment journey.

m2m has been implementing the Mentor Mother (MM) model in East and Central Uganda since 2010, originally under the Strengthening TB and HIV/AIDS Response in East Central Uganda (STAR-EC) Project[19]. Through this model, m2m trained, employed and empowered mothers living with HIV to work as MMs whose focus is on improving health education, peer support, family support, mother baby pair follow-up, supporting facility-community linkages and client retention in eMTCT/RMNCH at high volume and hard-to-reach health facilities in the region’s island and mainland fishing villages during the period of observation.

Through m2m’s Mentor Mother model, several community health care (CHC) programmes have been implemented across communities in Sub-Saharan countries with a view to improve health outcomes for pregnant women living with HIV and their babies. A recent scoping review identified inconsistencies in the outcomes of such CHC programmes [20]. It is also critical to periodically assess such programmes in the interest of best practices. The goal of this analysis was therefore to assess the effect of the m2m Mentor Mother model on retention of mother-child pair in HIV care and PMTCT in Uganda.

## Methods

### Setting

During the period of observation the Mentor Mother Programme was implemented across nine districts in East Central Uganda–Bugiri, Buyende, Iganga, Kaliro, Luuka, Mayuge, Namayingo and Namutumba—covering a population of approximately 3.1 million people (9% of Uganda’s total population). The health facilities that served as control were selected from districts in Mid-Western Uganda: Hoima, Kiboga, Kyankwanzi, Bullisa and Kibaale and were matched with those in the intervention areas by level of care and client load in each health facility.

The control group received the Government of Uganda usual standard of care for PMTCT services, the general PMTCT service package and family support groups which were introduced in 2013[21]. The intervention group received the standard of care for PMTCT as well as the psychosocial support provided by the Mentor Mothers from m2m. The study was conducted at four levels of care including hospitals, health centre IV, health centre III and health centre II. Health centre (HC) II provides outpatient services only at parish level and designated to serve a population of 500 people; HC III provides outpatient services, Maternity, general ward and laboratory services at sub county level and designated to serve a population of 20,000; HC IV provides outpatient services, maternity, ward, theatres, blood transfusion and Laboratory services at county level and designated to serve a population of 100,000 people; Hospitals provide all outpatient and inpatient services including ward, theatres, blood transfusion, X-ray and Laboratory services at district level and designated to serve a population of 500,000 people.

### Study design

We conducted a secondary analysis of data obtained from the m2m’s Uganda Mentor Mothers programme. The primary study was a quasi-experimental intervention targeted at women attending PMTCT clinics in Uganda between January 2011 and March 2014. Data for maternal and child health outcomes and impact indicators were obtained from patient records in the health facility. A health facility checklist was used to extract data from the facility records.

### Study population and sampling

The primary study population comprised of HIV-positive pregnant mother-child dyad. A two-stage sampling design was used to obtain a representative sample of HIV-positive women and their babies. The two-stage approach involved the selection of health facilities followed by the mother-child dyad. The sample size calculation assumed a design effect of two, 80% power, a type 1 error rate of 5% and a PMTCT enrolment rate of 78% in the intervention arm and 70% in the control. The enrolment assumptions are based on data from the Ugandan Ministry of Health’s 2012/2013 health management information system routine and unpublished data. The m2m / STAR-EC Mentor Mother Model in Uganda was implemented in 45 sites in 9 districts of East Central Uganda. The estimated sample size for health facilities at a 10% precision level was 31 facilities (for each study arm) using the formula n = N/(1+N(e)^2^ where e is precision level[22]. The exclusion criteria were a) mothers and babies whose records fell outside the January 2011 and March 2014 timeframe; and b) mothers and babies without complete records at the health facilities.

Health facilities were stratified by level of care in the intervention and control groups. A total of 1161 mother-baby pairs were selected among those enrolled in the m2m program between January 2011 and March 2014 while 1143 mother-baby pairs were selected as controls.

All hospitals and HC IVs were included in the study, the remaining health facilities were allocated to HC IIIs and HC IIs in proportion to the total number of targeted health facilities at different specific levels of care. The control health facilities were matched by level of care and client load in each health facility, with those selected in the intervention areas.

The entry point for selecting clients was the PMTCT clinic. Records of HIV-positive women who attended the PMTCT clinic between January 2011 and March 2014 were reviewed by a team of researchers. These records were found in patient registers at the sampled health facilities. The registers reviewed for the required information included antenatal care register, blood sample dispatch registers, delivery registers, ART registers and the postnatal registers.

### Measures

For the purpose of this analysis, we chose retention in HIV care as our primary outcome of interest. Retention in care for mother-baby pairs was measured as the proportion of mother-baby pairs enrolled for PMTCT services who remained in care at 6 weeks after birth, 6 weeks after cessation of breastfeeding and 18 months after birth expressed as percentages. We also assessed the effect of the programme on indicators such as percentage of HIV-positive women who were initiated on ART and were lost to follow-up; percentage of infants who received PCR testing at 6 weeks after birth; percentage of infants who received PCR testing at 6 weeks after Cessation of breast feeding; percentage of infants who received HIV testing at 18 months after birth.

### Data analysis

Frequencies for variables such as mother, parity and facility type were determined and the findings stratified by the study groups. Univariate and multivariate multilevel logistic regression analysis of all outcome indicators were conducted accounting for clustering (patients clustered in health facilities) at the facility level using Generalised Estimating Equation (GEE). Variables included in the multilevel model were age of mother, parity of mother, type/level of health facility and study arm (intervention and control arms). Additional sensitivity analysis on the outcome indicators comparing the control and intervention arms were conducted using propensity score matching: Calliper, nearest neighbour and inverse probability matching. For the calliper matching, a maximum distance between the intervention and matching control was fixed at 0.005 while for the nearest neighbour matching, mothers from the intervention arm were matched to an equal number of mothers from the control arm (1:1 matching). Inverse probability weighting was applied by taking the inverse of the propensity scores. Statistical analysis was conducted in STATA (StataCorp. 2017. *Stata Statistical Software*: *Release 15*. College Station, TX: StataCorp LLC).

### Ethical approval

Ethical approval was obtained from The AIDS Support organisation (TASO), a local institutional review committee and further registered and approved by the Uganda National Council of Science and Technology (UNCST). Permission was also obtained from the Chief Administrative Officers (CAO) in the respective districts to conduct the study at the health facilities. The ethics approval and government permission to conduct the study included capturing of clinical care and support information with no personal identification. Consequently, confidentiality and anonymity was maintained by not recording any identifying information (such as names, national identify numbers, telephone numbers or addresses on the patients’ folders) in the data extraction tool and database.

## Results

A total of 2304 HIV positive mother-baby pairs were included in the analysis, 1161 in the intervention and 1143 in the control group. Table 1 shows demographic characteristics of the 2304 participants. The majority of mothers in both arms were below 30 years of age (56.2% in intervention and 72.7% in controls). Most mothers attended health centre IV (47% in the intervention and 43.4% in the control group).

**Table 1:** Demographic characteristics of HIV positive women whose records were assessed.

Characteristic	Intervention n (%)	Control n (%)	P value#
**Overall**	1161 (50.4)	1143(49.6)	
**Age of mother (years)**			
≤30	649 (56.2)	816 (72.7)	<0.001
>30	505 (43.8)	306 (27.3)	
**Parity**			
≤3	530 (46.0)	617 (54.0)	0.042
>3	623 (54.0)	526 (46.0)	
**Facility Type**			
Health Centre II	79 (6.8)	36 (3.2)	<0.001
Health Centre III	330 (28.4)	445 (38.9)	
Health Centre IV	546 (47.0)	496 (43.4)	
Hospital	206 (17.7)	166 (14.5)	
**Entry to Care**			
Option A	712 (65.6)	566 (55.3)	0.093
Option B	374 (34.4)	458 (44.7)	

#-Chi square test adjusting for clustering.

### Mother and child retention on PMTCT across time points

**Fig 1:**
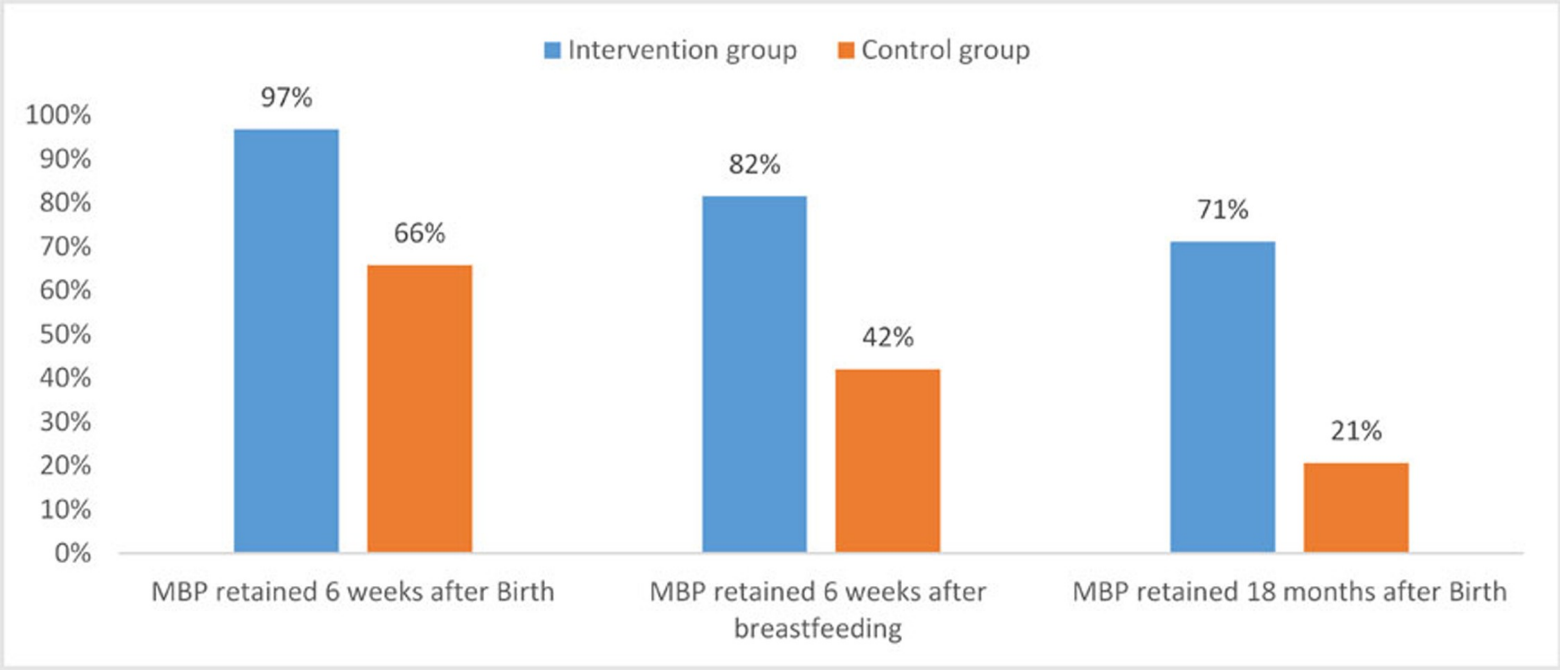
Proportion of mothers or mother-baby-pairs (MBP) remaining in case at key milestones.

Retention along the PMTCT cascade was higher for mother-baby pairs (MBP) in the intervention arm (n = 1161) compared to those in the control arm (n = 1143) (Fig 1), 97% vs 66% at 6 weeks after birth, 81.5% vs 42.0% at 6 weeks-after cessation of breastfeeding and 71.2% vs 20.6%, at 18 months after birth.

Analysis accounting for clustering at health facility level and adjusting for the effect of client age, parity, ART option which mothers were initiated on, and health facility type shows that women in the intervention arm were more likely to be retained in care 6 weeks-after birth (AOR 12.23, 95% CI 5.51, 27.14), 6 weeks-after cessation of breastfeeding (AOR 4.93, 95% CI 2.98, 8.12) and at 18 months after birth (AOR 8.65, 95% CI 4.80, 15.59) compared to women in control facilities (Table 2). Clustered analysis also shows that the retention for clients enrolled on Option B+ was significantly lower at 6 weeks-after cessation of breastfeeding (AOR 0.66, 95% CI 0.44, 1.00), and at 18 months-after birth (AOR 0.45, 95% CI 0.30, 0.67) compared to women enrolled in option A. This implied that women who were on Option B+ were 34% and 55% less likely to be retained in care than women on Option A at 6 weeks after cessation of breastfeeding and at 18 months after birth respectively (Table 2). Furthermore, there was generally an increase in retention among individuals aged 30 years or older in both control and intervention arms at 6 weeks-after birth, 6 weeks after cessation of breastfeeding and at 18 months 18 months after birth (Table 2).

**Table 2:** Adjusted clustered analysis (client retention in the program over the 3 time points).

Indicator	6 weeks after Birth AOR (95% CI)	6 weeks after Cessation of breastfeeding AOR (95% CI)	18 months after Birth AOR (95% CI)
**Arm**			
Control	Ref	Ref	ref
Intervention	12.23 (5.51, 27.14)*	4.93 (2.98, 8.12)*	8.65 (4.80, 15.59)*
**Age of mother (years)**			
≤30	Ref	Ref	ref
>30	1.34 (1.05, 1.69)*	1.28 (1.07, 1.54)*	1.39 (1.16, 1.66)*
**Parity**			
≤3	Ref	Ref	ref
>3	0.97 (0.73, 1.27)	0.94 (0.75, 1.17)	0.93 (0.75, 1.14)
**Facility Type**			
HC II	Ref	Ref	ref
HC III	0.27 (0.01, 16.41)	0.56 (0.21, 1.51)	0.75 (0.31, 1.78)
HC IV	0.20 (0.00, 13.60)	0.36 (0.14, 0.96)*	0.66 (0.27, 1.58)
Hospital	0.54 (0.01, 42.24)	0.82 (0.28, 2.38)	1.02 (0.40, 2.60)
**Entry to Care**			
Option A	Ref	Ref	ref
Option B	1.06 (0.72, 1.56)	0.66 (0.44, 1.00)*	0.45 (0.30, 0.67)*

*significant at p<0.05, AOR: adjusted odds ratio.

The results in Table 3 mostly show no significant difference in proportions of retained mother-baby-pairs who received the prescribed PMTCT services in the intervention and control groups at the different time points along the PMTCT cascade. For instance, 96.2% of retained HIV positive pregnant women got PCR test done for their infant at 6 weeks after birth at the intervention sites versus 86.8% in the control group; 70.9% of retained HIV positive pregnant women in the intervention sites got PCR test done for their infant at 6 weeks after Cessation of breast feeding versus 69.6% of retained control group clients; and the 71.7% of retained HIV positive pregnant women in the intervention sites who got PCR test done for the infant at 18 months after birth versus 68.9% of retained control group clients who received similar service. The similar proportion of women who received the prescribed PMTCT interventions at different stages should be interpreted against significantly higher number of mother-baby-pairs retained in the intervention group when compared to numbers retained in the control group at the different critical points (Fig 1 and Table 2). In furtherance of this observation, the risk of getting lost to follow-up in the intervention group among women that were initiated on ART was consistently lower than that of the controls (Table 3) across the calliper (OR: 0.05, 95% CI: 0.02, 0.15), nearest neighbour (OR: 0.04, 95% CI: 0.01, 0.13) and inverse probability matching (OR: 0.03, 95% CI: 0.01, 0.11) methods.

**Table 3:** Multivariate analysis after calliper matching, nearest neighbour and inverse probability matching.

Outcome Indicator	Calliper Matching AOR (95% CI)	Nearest neighbour AOR (95% CI)	Inverse Probability Matching AOR (95% CI)
Percentage of HIV positive women who were initiated on ART and got lost to follow-up (Missed 3 consecutive appointments) within 12 months of ART initiation.	0.05 (0.02, 0.15)*	0.04 (0.01, 0.13)*	0.03 (0.01, 0.11)*
Percentage of infants who received PCR testing at 6 weeks after birth	2.70 (0.98, 7.45)	2.51 (0.93, 6.79)	2.58 (0.93, 7.20)
Percentage of infants who received PCR testing at 6 weeks after Cessation of breast feeding	0.50 (0.18, 1.41)	0.48 (0.17, 1.39)	0.54 (0.17, 1.72)
Percentage of infants who received HIV testing at 18 months after birth	0.61 (0.15, 2.47)	0.66 (0.16, 2.70)	0.71 (0.18, 2.72)

*significant at p<0.05, AOR: adjusted odds ratio.

## Discussion

This analysis underscores the effectiveness of the mothers2mothers Ugandan Mentor Mother programme, demonstrated in the significantly better retention of mothers and their children at the intervention sites when compared to mother-child pairs at the control sites. This supposition is also grounded on the established benefits of clients’ retention on PMTCT outcomes [23]. This finding is strongly demonstrated by the higher number of retain intervention group members accessing the prescribed PMTCT services along the cascade. Although our study findings did not corroborate higher retention with higher uptake of infant services, prior studies have shown that the longer a mother stays in care with her child, the more opportunity for early diagnosis and treatment for the child, and this creates opportunity for the mother to receive appropriate and adequate treatment and acquire skills on how to protect her baby (5,8,9).

The highlight of the superiority of the m2m Mentor Mother Programme was demonstrated through the 71% vs 21% mother and child retention rates at 18 months along the PMTCT cascade at the intervention and control sites respectively. In addition, 97% of the Mother-baby pair were retained 6 weeks after birth and 82% retained 6 weeks after breastfeeding compared to the 66% and 42% respectively that was reported in the control arm. Our findings further showed a higher percentage of HIV positive mothers at the intervention sites who remained active on ART 12 months after ART initiation when compared to women at the control sites. These findings align our data with data from studies reporting a connection between retention in HIV care and delivery of PMTCT services by Lay Health Worker [24–26]. The benefits of the m2m Mentor Mother programmes as documented by our study are hallmarks of an effective PMTCT programme.

Although, our analysis found that there was a decreasing trend in retention rates from 6 weeks after birth until 18 months after birth in both intervention and control arms, the outcome was significantly better for the participants in the intervention arm. However, loss of clients along the PMTCT cascade is concerning because it is also a crucial period for ART initiation in HIV-infected infants [27]. Similar trends have been observed in other studies where retention was high during pregnancy but decreased rapidly in the postnatal period [28]. Other studies have reported cumulative retention rates of up to 80% six months postpartum [27–29]. Evaluation of control sites in our study at best reported almost 40% attrition in mother-child pairs and at its lowest point reported almost 80% attrition. The observed high and persistent attrition particularly at the government standard of care (control) sites mirrors similar and recent surveys conducted in Uganda [30,31].

The above observations add to existing evidence on the value of community health workers in promoting MBP retention along the PMTCT cascade [32–34]. Early infant diagnosis is not only critical for preventing HIV in exposed infants but also for reducing mortality in those infants who become infected [34,35]. One has to also be cognisant of the fact that the rate of infant testing and receipt of the results is also determined by the health system factors rather than the behaviour of the mothers/caregivers. For instance, the ability of the health system to provide prescribed PMTCT services to similar proportion of retained patients, at different time points in the intervention and control groups, in spite of their differences in numbers, is underscored and emphasize the importance of patient retention. To fully appreciate this point, our results showed that about 70% of the approximately 820 mother-baby-pair retained at 18 months in the intervention group received the final infant test compared to the 69% of the 240 mother-baby-pair retained at 18 months in the control group who received the final infant test.

This study is not without limitations. This was a secondary analysis of health system data and typical of such data from most low and middle income countries, some of the indicators observed during follow-up had missing data. Clustered GEE analysis was fitted in this data but was impacted by sparse data for some indicators leading to wide confidence bands for odds ratios. Despite these challenges, propensity score matching was fitted to bring similarity between groups and the findings were reassuring in terms of an assessment of reliability of the analysis. Lastly, we could not establish from the data if MBP were indeed lost to follow-up or deceased.

## Conclusions

The m2m Ugandan Mentor Mother Programme was effective in improving retention of mother-child pair in HIV care and PMTCT. The identification and adoption of the drivers of success in the m2m Uganda Mentor Mother Programme could guide or complement effective improvement of the Ugandan standard of care programme performance. The findings also offer many lessons to inform implementation of similar lay health worker programs targeting MTCT of HIV in sub-Saharan Africa. Adaptation of this peer-to-peer approach would complement retention in care strategies and could be adapted into other care programs targeting key populations such as adolescents, males, female sex workers, inmates and drug users.

## Supporting information

S1 File Data for this project. (XLS)
